# Impact of diaphragm function parameters on balance maintenance

**DOI:** 10.1371/journal.pone.0208697

**Published:** 2018-12-28

**Authors:** Janusz Kocjan, Bożena Gzik-Zroska, Katarzyna Nowakowska, Michał Burkacki, Sławomir Suchoń, Robert Michnik, Damian Czyżewski, Mariusz Adamek

**Affiliations:** 1 Chair and Department of Thoracic Surgery, Faculty of Medicine and Dentistry, Medical University of Silesia, Katowice, Poland; 2 Department of Biomaterials and Medical Devices Engineering, Faculty of Biomedical Engineering, Silesian University of Technology, Zabrze, Poland; 3 Department of Biomechatronics, Faculty of Biomedical Engineering, Silesian University of Technology, Zabrze, Poland; University of Ontario Institute of Technology, CANADA

## Abstract

The diaphragm is well known for its role as the principal muscle of respiration. However, according to previous studies, its role is multifactorial, from breathing through pain perception, regulation of emotional sphere, collaborating in gastroesophageal functions, facilitating the venous and lymphatic return, to an essential role in the maintenance of lumbar spine stability. The purpose of the study was to examine whether diaphragm function parameters (thickness and range of motion) are associated with static balance maintenance. A total of 142 participants were examined and divided into three groups: G1—patients qualified for lung resection due to cancer; G2 –patients after lobe resection; G3 –healthy subjects. Diaphragm thickness and excursion was measured using ultrasonography. Stabilometric parameters of balance were assessed by Zebris FDM-S platform. Greater diaphragm thickening during active breathing and diaphragm thickness fraction were associated with better static balance parameters. Limitation of diaphragm motion during quiet breathing and deep breathing was linked to balance disorders. There was no correlation between diaphragm muscle excursion during sniff maneuvers and balance parameters. Deterioration of diaphragm function observed after thoracic surgery was closely related with deterioration of balance maintenance. Impairment of diaphragm function manifested by decrease of muscle thickness and movement restriction is strongly associated with balance disorders in a clinical sample and among healthy subjects.

## Introduction

Maintenance of balance, also referred to as postural control, is considered a crucial component in many independent functional activities of daily living, from simple activities, such as standing (static balance), to more complex activities, such as walking or turning (dynamic balance) [1]. Despite the fact that the term is frequently used in a wide variety of clinical specialties, there is no universally accepted definition of balance or related terms. Nowadays, it is believed that postural control is the capacity for actively restoring the typical body posture lost due to destabilizing factors [2]. However, a certain amount of sway is essential and inevitable due to small perturbations within the body (e.g., breathing cycle), or from external triggers (e.g., visual distortions); therefore from a biomechanical perspective, this term refers to an individual's ability to minimize movement from the center of gravity on a basal plane[3–5]. Irrespective of the existence of various definitions, there is general agreement that maintaining balance requires the coordination of inputs from multiple sensory systems including: the vestibular system, the somatosensory system, and the visual system [6,7]. The relative contribution of each sensory system can be changed depending on environmental conditions and modulated by motor task [8–11].

The diaphragm muscle is both, the physical barrier that separates the thoracic cavity from the abdominal cavity, and the primary muscle of ventilation [12]. However, recent studies revealed that the diaphragm has other important functions in addition to ventilation. It is essential for cardiac function, venous and lymphatic return, gastroesophageal functions (emesis, swallowing, vomiting), regulation of emotional states, and threshold of pain. The diaphragm also has properties related to the maintenance of lumbar spinal stability [13–16]. This postural function refers to the contribution of the diaphragm to human trunk stabilization during tasks that require repetitive movement. Even though the diaphragm has a direct anatomical connection to the spine via its crural fibers, it cannot move the trunk voluntary. However, its contraction contributes to spinal stability via an increase of intraabdominal pressure (IAP). In addition, diaphragmatic contraction increases stability of the trunk by minimizing displacement of the abdominal contents into the thorax, maintaining a hoop-like geometry of the abdominal muscles, which increase spinal stability through tension in the thoracolumbar fascia. The trunk stabilization occurs prior (forward feedback) to the initiation of any voluntary movements of the limbs. This dual function of the diaphragm (ventilation and posture) is performed simultaneously, and is independent of the phase of respiration, which suggests that the response may be preprogrammed by the central nervous system [16–18].

We hypothesize that the postural function of the diaphragm can have a much wider functionality beyond spinal stability. There is evidence that the diaphragm is activated during tasks that challenge the stability of the spine, and that it is also activated in the preparatory phase of movements that induce postural instability. It is reasonable to suppose that the proper stabilization of the lumbar spine will directly affect the global equilibrium of a human. However, there is a lack of study directly designed to test this hypothesis. The objective of this study was to investigate whether diaphragm function parameters are associated with static balance parameters. We present here a hypothesis that the diaphragm thickness and diaphragm thickness fraction are reduced among subjects with worse balance parameters. In addition, we expected that diaphragm excursion during breathing maneuvers was lower in participants with balance disorders.

## Material and methods

A randomized controlled study with blind assessment was carried out at the Thoracic Surgery Department of The Professor S. Szyszko Teaching Hospital No 1 in Zabrze. The study was approved by the Ethics Committee of Silesian Medical University (Decision number: KNW/0022/KB1/77/I/16) and conducted in accordance with the amended Declaration of Helsinki. An information sheet was given to patients on admission to the hospital ward. At the bedside, each patient was given a verbal explanation of the nature of the study by the investigator. If agreed to participate in the study, an informed consent was signed. Volunteers were assessed from 30/09/2016 to 20/07/2017.

### Subjects

A total of 102 participants were examined (52.3% males, 47.7% females; mean group age: 49.6 ± 9.24; age range: 24–78 years). The clinical sample consisted of 62 patients (61.3% males, 38.7% females) with mean age 62.06 ± 10.44 years (range: 42–78). Recruitment occurred in 2 phases. In the first phase, a sample of 62 patients was chosen. These patients qualified for lobe resection due to lung cancer when they were evaluated before surgery. In the second phase, selected patients (n = 40; 62,5% males, 37,5% females; mean group age: 63.74 ± 11.87 years; age range: 42–78 years; 57.5% have undergone classic thoracotomy, 42.5% have undergone VATS) who were after lobe resection and fulfilled the inclusion criteria, were assessed a second time 3–5 days after the surgical procedure. Overall, 102 measurements were performed. This allowed the assessment of the discussed issue in two different groups of patients: group 1 –patients with diagnosis of lung cancer, and group 2 –patients after lobe resection, which also allowed the evaluation of whether the impaired function of the diaphragm after lung resection affects the equilibrium parameters. Group 3 (non-clinical control group) included 40 healthy individuals (40% males, 60% females, mean group age: 25.82 ± 2.26; age range: 24–46) who were students at a Medical University, University of Technology and medical staff, and were not diagnosed with any chronic diseases.

The inclusion criteria of the patient group was: age over 18 years, a written consent of the patient to participate in the study, diagnosis of lung tumor, eligibility for lobectomy, and lack of coexisting diseases which may contribute to imbalance. The following criteria for exclusion from the study were used: inability to understand explanations or verbal instruction, a clear view of the diaphragm could not be obtained during the testing procedure, a history of spontaneous falls, gait disturbances, impaired walking balance, vision impairment, diabetes mellitus, advanced motor disorders, impaired mobility, orthostatic hypotension, and intellectual disability. Poor physical condition after surgery and drains in thoracic cavity were also exclusion criteria during qualification for the second measurement.

### Balance measurement

Zebris FDM-S platform was used to assess stabilometric parameters of balance. The device is a force-measuring platform with built-in capacitive force sensors enabling the measurement and analyses of force distribution under the feet (dimensions: 69 x 40 x 2.1 cm, Sensor surface: 54 x 33 cm, number of sensors: 2,560, sampling rate: 120 Hz).

In assessing static stability, patients stood barefoot in a natural and relaxed position, with their arms by their sides, and with both heels parallel to each other. During the assessment of stability, subjects were asked to stay as still as possible, while one of the investigators stood behind them to prevent any falls. Volunteers completed the static balance task under 2 sensory conditions: 1. eyes open and facing toward the target, 1,5m away (OE), and 2. eyes closed (CE). Each task took 30 seconds, with a 1 minute break between each measurement.

The following stabilometric quantitative parameters were considered: path (total length of center of gravity displacement), ellipse (area in which the center of gravity oscillates), left and right side loading, back and fore side loading both in open (OE) and closed (CE) eyes conditions.

### Ultrasound measurement

Assessments of the diaphragm were carried out using an ultrasound scanner ALOKA 10 with a multi-frequency convex probe. Patients were evaluated in the supine position, which was preferred, as it is more comfortable for the patient, shows less variability, minimizes side to side variation, and allows greater excursion of the diaphragm [19]. The supine position also exaggerates any paradoxical movement and limits any compensatory active expiration by the anterior abdominal wall which may mask paralysis [20]. The first ultrasonographic measurement was performed within 2h after admission to the thoracic surgery ward. Second recording was acquired 2–5 days post surgery, after the drains were removed from the thoracic cavity. All evaluations were made by the same trained investigator who was blinded to the status of the balance parameters of each patient.

### Measurement of diaphragm thickness by B mode ultrasound

For evaluation of diaphragmatic thickness parameters, the diaphragm was visualized by placing the high frequency 7MHz transducer at the zone of apposition perpendicular to the chest wall, between 7th and 9th intercostal space, in the anterior axillary lines inferiorly to the costophrenic angle. The diaphragm was observed as a structure made of three distinct layers: a non-echogenic central layer bordered by two echogenic layers, the diaphragmatic pleura and the peritoneal membrane. Once a clear image of the diaphragm was obtained, the diaphragm thickness was estimated as the vertical distance between the last set of parallel lines on the image [19,21].

Diaphragm thickness was measured in two different efforts: maximal inspiratory and maximal expiratory. Measurements of diaphragm thickness were obtainied at end expiration(the patient was instructed to perform breathing to total lung capacity (TLC) and then exhale to end expiration.) Measurements of the diaphragm thickness at TLC were obtained by asking participants to take a maximal inspiration starting from the end of residual volume. An index of diaphragmatic thickening (DTF, diaphragm thickening fraction) was calculated using the formula: DTF = thickness at end-inspiration—thickness at end-expiration/thickness at end-expiration x 100% [19,21]. Diaphragm thickening of less than 20% is proposed to be consistent with paralysis [22].

According to a previously validated method, only the right hemidiaphragm was used to measure the thickness of the diaphragm, as literature has shown that there is no significant difference in thickness on the left side [19,21]. Measurements were taken three times and mean values were used for analysis.

### Measurement of diaphragm movement by M mode ultrasound

For evaluation of diaphragmatic excursion a low frequency 4MHz curvilinear transducer was placed between the mid-clavicular and anterior axillary lines, directed medially, cranially and dorsally to visualize the posterior third of the right diaphragm, approximately 5 cm lateral to the inferior vena cava foramen. The right diaphragm dome was visualized behind the liver on a subcostal view between the anterior and midaxillary lines, where the ultrasound beam is aligned perpendicularly to the posterior part of the diaphragm. A similar recording was performed on a left subcostal view after visualizing the posterior part of the left diaphragm behind the spleen. During M-mode imaging, the normally functioning diaphragm was represented as an echogenic line that moves freely during inspiration and expiration. The motion of hemidiaphragm was captured during normal quiet breathing (QB), deep breathing (DB) and sniff maneuvere. Inspiration was considered as an upward motion in the M-mode tracing. The amplitude of excursion was measured on the vertical axis of the tracing from the baseline to the point of the peak of the tracing line on the graph. For each maneuver, three satisfactory readings were performed for each side and the average value was taken [19,21].

### Statistical analysis

Statistical analyses were performed using STATISCTICA StatSoft version 12.0. All data are expressed as mean (M) ± Standard Deviations (SD) of the mean. Shapiro-Wilk test was used to analyze the normality and homogeneity of data variance. The relationship between diaphragm function and balance parameters was assessed using the Pearson correlation test. Within the three groups of participants, comparisons in terms of diaphragmatic functional parameters and balance parameters were made using Wilcoxon signed-rank test (for preoperative and postoperative patients) and t-student test for the independent group (for clinical groups and healthy subjects). A P-value of less than 0.05 was deemed to be statistically significant.

## Results

We found statistically significant differences in the diaphragm thickness and motion in patients undergoing lung resection, compared to preoperative baseline data. Similar differences (except diaphragm exhalation thickness) were observed by comparing the control subjects to patients undergoing lung resection. Between group 1 and group 3, differences were reported only in the case of exhalation thickness and DTF values. Descriptive statistics of diaphragm functional parameters and differences between groups are presented in Table 1.

**Table 1:** Diaphragm functional parameters: means, standard deviations and 95% Confidence Interval.

Variables	Group 1	Group 2	Group 3	P value
Thickness Ins [cm]	0.30±0.05 (0.28–0.31)	0.27 ± 0.04 (0.26–0.29)	0.32±0.08 (0.29–0.35)	**1–2: p<0.000**; 1–3: p = 0.207; **2–3: p<0.001**
Thickness Exp [cm]	0.23±0.04 (0.21–0.24)	0.21 ± 0.07 (0.18–0.23)	0.20±0.01 (0.20–0.21)	**1–2: p = 0.009**; **1–3: p = 0.015;** 2–3: p = 0.224
DTF [%]	35.33±21.22 (29.43–41.24)	24.17 ± 13.85 (18.99–29.34)	56.77±42.23 (41.00–72.54)	**1–2: p<0.000**; **1–3: p = 0.003**; **2–3: p<0.001**
QB right [cm]	1.81±0.43 (1.69–1.93)	1.47 ± 0.41 (1.36–1.58)	1.74±0.21 (1.66–1.82)	**1–2: p<0.000**; 1–3: p<0.088; **2–3: p<0.001**
DB right [cm]	4.74±1.28 (4.38–5.10)	3.84 ± 0.92 (3.49–4.19)	5.21±1.62 (4.61–5.82)	**1–2: p = 0,000**; 1–3: p = 0.149; **2–3: p<0,001**
Sniff right [cm]	2.43±0.55 (2.28–2.59)	2.14 ± 0.40 (1.99–2.23)	2.48±0.41 (2.26–2.59)	**1–2: p<0,000**; 1–3: p = 0.671; **2–3: p<0,001**
QB left [cm]	1.89±0.44 (1.77–2.12)	1.45 ± 0.31 (1.32–1.62)	1.83±0.21 (1.75–1.91)	**1–2: p = 0,000**; 1–3: p<0.526 **2–3: p<0,001**
DB left [cm]	4.98±1.30 (4.61–5.34)	3.72 ± 0.91 (3.58–4.07)	5.37±1.64 (4.74–5.98)	**1–2: p<0,000**; 1–3: p = 0.237; **2–3: p<0.001**
Sniff left [cm]	2.60±0.57 (2.44–2.76)	2.08 ± 0.36 (1.86–2.20)	2.72±0.32 (2.55–2.89)	**1–2: p<0,000**; 1–3: p = 0.259 **2–3: p<0.001**

Ins = Inspiratory; Exp = expiratory; DTF = Diaphragm Thickness Fraction; QB = quiet breathing; DB = deep breathing

Table 2 contains data on static equilibrium parameters and values of groups comparisons. Presented findings point to a postoperative increase of mean values of path length and ellipse area after lobectomy in comparison to preoperative data, only with the eyes open. Differences between healthy subjects and clinical group were demonstrated in both, open and closed eyes conditions.

**Table 2:** Balance parameters: means, standard deviations and 95% Confidence Interval.

Variables	Group 1	Group 2	Group 3	P value
Lenght OE [mm]	398.95±110.37 (371.38–426.52)	458.24±193.94 (397.03–519.46)	338.15±59.53 (315.92–360.38)	**1–2: p = 0.023**; **1–3: p = 0.003;** **2–3: p<0.001**
Ellipse OE [mm^2^]	67.70±62.74 (52.03–83.38)	120.62±152.40 (72.49–168.70)	39.49±25.84 (29.83–49.14)	1–2: p = **0.002**; **1–3: p = 0.008;** **2–3: p<0.001**
Lenght CE [mm]	544.97±280.82 (474.82–615.11)	591.70±263.30 (506.34–677.05)	429.92±103.18 (391.39–468.45)	1–2: p = 0.154; **1–3: p = 0.013;** **2–3: p<0.001**
Ellipse CE [mm^2^]	96.12±95.51 (72.27–119.98)	115.16±92.25 (85.65–144.66)	48.4±34.30 (35.58–61.21)	1–2: p = 0.108; **1–3: p = 0.001;** **2–3: p<0.001**
Left side load OE [%]	49.42±6.42 (47.82–51.02)	48.39±5.61 (46.62–50.16)	50.94±4.16 (49.38–52.49)	1–2: p = 0.937; 1–3: p = 0.240; 2–3: p = 0.946
Right side load OE [%]	50.57±6.42 (48.97–52.17)	64.56±22.14 (58.63–70.49)	49.05±4.16 (47.50–50.61)	1–2: p = **0.001**; 1–3: p = 0.240; **2–3: p<0.001**
Module (L-R side) OE [%]	10.15±7.99 (8.16–12.15)	29.67±39.66 (19.76–39.58)	6.38±5.56 (4.30–8.45)	**1–2: p = 0.020**; **1–3: p = 0.009;** **2–3: p<0.001**
Left side load CE [%]	49.82±5.47 (48.45–51.19)	49.11±6.20 (47.12–51.09)	50.59±4.22 (49.01–52.16)	1–2: p = 0.868; 1–3: p = 0.558; 2–3: p = 0.443
Right side load CE [%]	50.16±5.47 (48.80–51.53)	64.91±22.98 (58.76–71.07)	49.41±4.22 (47.83–50.98)	**1–2: p<0.001**; 1–3: p = 0.561; **2–3: p<0.001**
Module (L-R side) CE [%]	8.55±6.76 (6.86–10.24)	31.45±40.39 (21.36–41.54)	6.11±5.85 (3.92–8.29)	**1–2: p<0.001**; 1–3: p = 0.054; **2–3: p<0.001**
Back OE [%]	55.31±10.35 (52.72–57.90)	41.46±15.99 (37.18–45.75)	56.03±11.20 (51.85–60.22)	**1–2: p = 0.005**; 1–3:p = 0.570; **2–3: p<0.001**
Fore OE [%]	44.98±10.33 (42.40–47.56)	58.53±26.92 (51.32–65.74)	43.96±11.20 (39.77–48.14)	1–2: p = 0.224; 1–3: p = 0.573; 2–3: p = 0.113
Back CE [%]	53.60±8.96 (51.37–55.84)	39.91±15.64 (35.72–44.11)	55.55±8.75 (52.28–58.82)	**1–2: p<0.001**;1–3: p = 0.408; **2–3: p<0.001**
Fore CE [%]	46.39±8.96 (44.15–48.62)	60.10±26.59 (52.98–67.23)	44.44±8.75 (41.17–47.71)	**1–2: p<0.001;** 1–3: p = 0.408; **2–3: p<0.001**

OE = open eyes; CE = close eyes; Back = load of back side of body; Fore = load of fore side of body

Correlations of diaphragm thickness and diaphragm movement with length of path and area of ellipse, both with open and closed eyes, were reported. Detailed results are given in Table 3.

**Table 3:** Correlations between diaphragm functional parameters and selected balance parameters.

Variables	Group	Path OE	Ellipse OE	Path CE	Ellipse CE
Thickness Ins	1	-0.214*	-0.157*	-0.289*	-0.173
	2	-0.229*	-0.217*	-0.296*	-0.319*
	3	-0.555***	-0.579***	-0.602***	-0.498**
Thickness Exp	1	0.133	0.440***	-0.063	0.087
	2	0.273	0.117	0.133	0.135
	3	0.158	0.255	0.290	0.150
DTF	1	-0.332**	-0.331*	-0.285*	-0.280*
	2	-0.573***	-0.375*	-0.502**	-0.489**
	3	-0.621***	-0.576***	-0.633***	-0.513***
QB right	1	-0.296*	-0.322*	-0.257*	-0.457***
	2	-0.325*	-0.216	-0.443**	-0.451**
	3	-0.512***	-0.528***	-0.536***	-0.515***
DB right	1	-0.433**	-0.362**	-0.291*	-0.426**
	2	-0.528**	-0.342*	-0.329*	-0.458**
	3	-0.622***	-0.631***	-0.633***	-0.624***
Sniff right	1	-0.186	-0.184	0.032	-0.245
	2	-0.176	-0.234	-0.197	-0.232
	3	-0.355	-0.364	-0.337	-0.348
QB left	1	-0.295*	-0.314*	-0.230	-0.456***
	2	-0.349*	-0.234	-0.435*	-0.421*
	3	-0.516***	-0.532***	-0.541***	-0.522***
DB left	1	-0.446***	-0.376**	-0.310*	-0.441**
	2	-0.537***	-0.350*	-0.407*	-0.464**
	3	-0.624***	-0.636***	-0.639***	-0.631***
Sniff left	1	-0.211	-0.216	0.003	-0.307*
	2	-0.180	-0.176	-0.190	-0.195
	3	-0,318	-0.299	-0.308	-0.315

Ins = Inspiratory; Exp = expiratory; DTF = Diaphragm Thickness Fraction; QB = quiet breathing; DB = deep breathing; OE = open eyes; CE = close eyes;

* = p<0,05;

** = p<0,01;

*** = p<0,001

**Fig 1:**
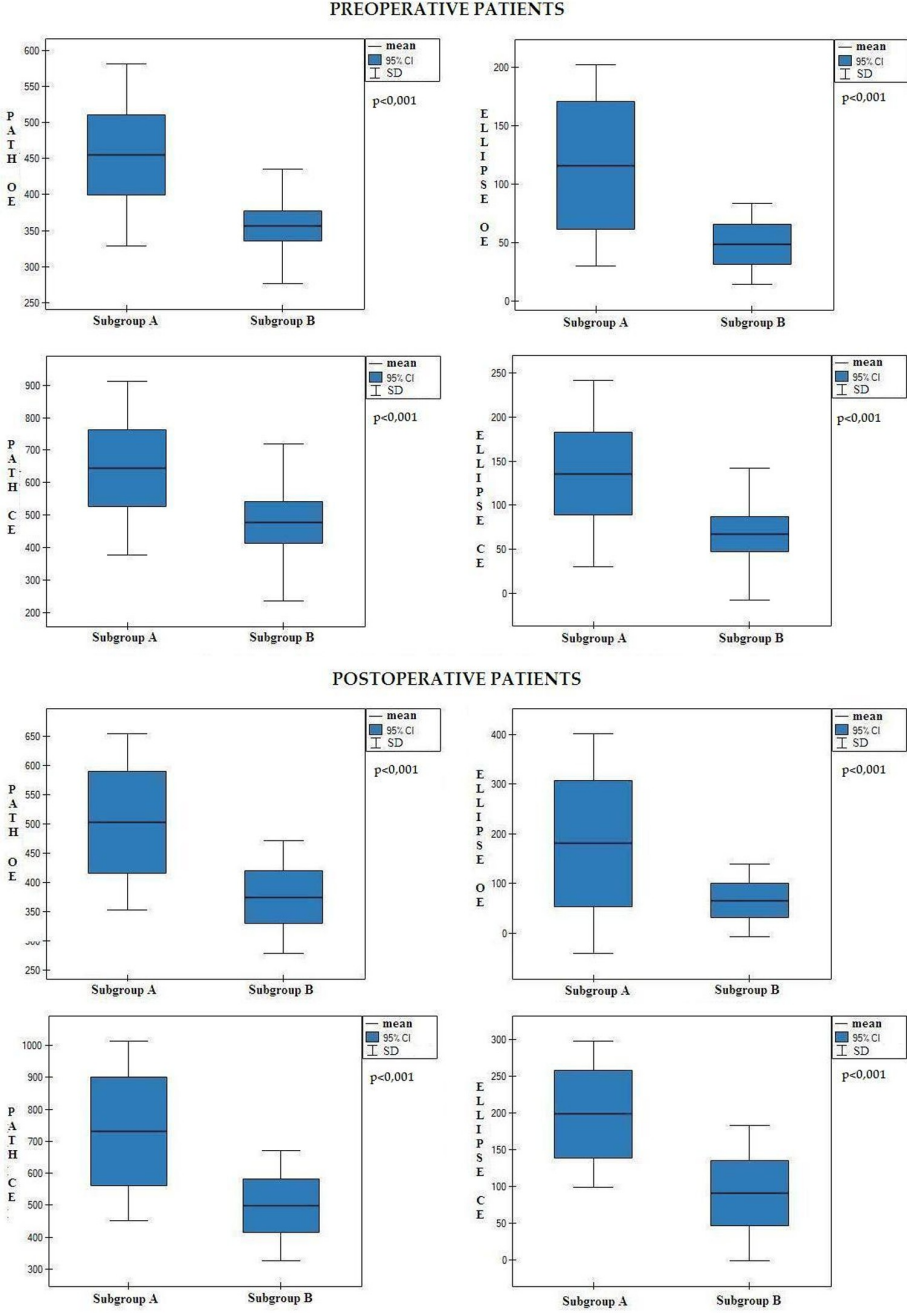
Preoperative and postoperative values of path lenght and ellipse field. OE: open eyes; CE: close eyes; Subgroup A: diaphragm paralysis; Subgroup B: normal function of diaphragm.

**Fig 2:**
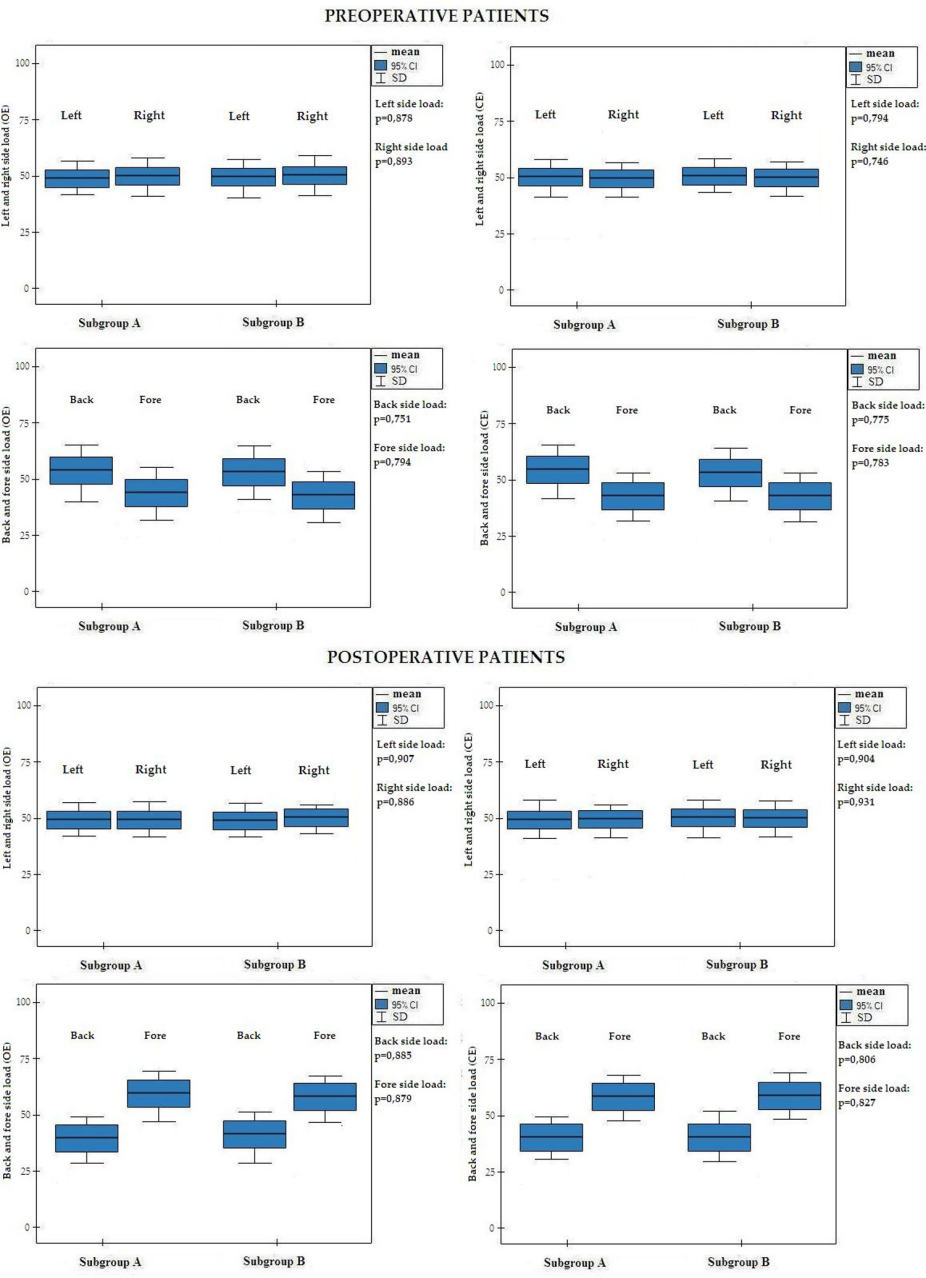
Preoperative and postoperative values of back, fore, left and right side loading. OE: open eyes; CE: close eyes; Subgroup A: diaphragm paralysis; Subgroup B: normal function of diaphragm.

DTF values less than 20%, indicating diaphragm paralysis, were found among 30.6% (n = 19) preoperative patients and among 42.5% postoperative (n = 17) patients.The DTF parameters were within normal limits in all patients in the control group. Next, subjects were divided into two subgroups as a criterion assuming the DTF values (Subgroup A: diaphragm paralysis; Subgroup B: normal diaphragm function). Both preoperative and postoperative patients with diaphragm paralysis had statistically significant higher values of ellipse field and path length with eyes open, as well as with eyes closed, as compared to patients with physiological DTF values. Detailed results for the preoperative and postoperative groups are presented in Figs 1 and 2.

## Discussion

The main purpose of the study was to address the question of whether the diaphragm muscle parameters are linked with the parameters of static balance maintenance. We found that greater values of diaphragm inspiratory thickness, diaphragm thickness fraction and diaphragm movement during quiet breathing and deep breathing are associated with better equilibrium parameters in the form of shorter path length and smaller ellipse field, both with eyes open, and with disabled visual control. We demonstrated the occurrence of this relationship among patients with diagnosis of lung cancer and patients undergoing lung resection, as well as among healthy population. Furthermore, we reported that the deterioration of diaphragm function observed after thoracic surgery is closely related to the deterioration of balance maintenance.

Mraz et al., observed a change of the center of gravity position and a decrease of postural stability in patients after thoracic surgery. The authors conclude that this is related to the violation of the muscular corset during the classic thoracotomy and the post-operative occurrence of pain. The pain after surgery may cause forced and incorrect position of the body subconsciously protect themselves from pain [23]. In the present study, we reported that detorioration of balance maintenance observed after thoracic surgery is closely related to detorioration of diaphragm function. Analizing data presented in Table 2, we observed a postoperative increase of mean values of path length and ellipse area after lobectomy in comparison to baseline preoperative data—similar to diaphragm parameters results. Noteworthy is the fact that resection of the lung lobe causes the balance disorders in the field of load transfer through the forefoot and the rearfoot. Patients qualified for surgery, as well as those in the control group, were characterized by a higher forefoot load, unlike to a patients undergoing surgical procedures with a higher load on rearfoot.

In our opinion, four possible theoretical explanations of this phenomenon exist. These are the afferent conduction of the vagus nerve pathway and the existence of the vestibulo-autonomic reflex; the presence of specialized mechanoreceptors (proprioceptors) in the diaphragm cruras and their possible stimulation by intra-abdominal pressure; diaphragm muscle fatigue; and the phrenic nerve pathway. We describe each possible mechanism below.

### 1. The role of the vagus nerve

There is a strong relationship between neural activity in the vestibular system and activity of the autonomic nervous system. Reactions such as nausea, vomiting, or tachycardia clearly demonstrate the existence of "vestibulo-autonomic reflexes" [24]. Previous study reported the direct connections between the vestibular nuclei and two brain stem regions that mediate autonomic functions, nucleus tractus solitarius, and the dorsal motor nucleus of the vagus nerve [25]. However, interactions between vestibular and autonomic system have been considered as a bidirectional pathways of information. [26].

Diaphragmatic breathing is part of a feedback loop that improves vagal tone by stimulating the relaxation response of the parasympathetic nervous system [27]. Change in breathing pattern from diaphragmatic to thoracic leads to overactivity of the sympathetic nervous system. We hypothesized that in consequence of "switch" disorder between these two systems, increased afferentation from the vagus nerve to the vestibular system can result in postural unsteadiness.

Additionally, thoracic breathing contributes to decreased levels of carbon dioxide (CO2) in the bloodstream and often leads to a degree of hyperventilation [28]. Resulting respiratory alkalosis creates a state of sympathetic dominance [29]. Two previous studies documented that hyperventilation affects balance and frequently interferes with somatosensory mechanisms and central processes mediating vestibular compensation. Sakellari et al., found that healthy subjects become posturally unstable, with large amplitude and low frequency body sway movements, when in hypocapnia [30]. Patients with severe bilateral loss of peripheral vestibular function ('labyrinthine defective subjects'‘) became equally unstable during hyperventilation, suggesting that the unsteadiness was more likely to be mediated by non-vestibular than by direct vestibular mechanisms [31]. In the present study we noted that 30% of preoperative patients and 42,5% of postoperative patients have diaphragm paralysis identified as a diaphragm thickness fraction less than 20%. We suppose that due to limitations of diaphragm movement efficiency, the shallow chest breathing dominate among this patients and in consequence may lead to a hyperventilation.

The vagus nerve also has the ability to transmit visceral pain to the supraspinal centers due to a retrograde transport of bio-chemicals through the nerve, and it contributes to the formation and maintenance of the central pain memory modulating inhibitory descendant pathways to nociceptive areas in the spinal cord. In recent study, Bordoni et al., suggested that, if the sympathetic nerves are compressed in the region of the diaphragm, their function and morphology can change limiting its antinociceptive and antiinflammatory ability, causing nociceptive afferents and negatively affecting the innervated tissues [15]. We speculate that similar mechanism may also occur after thoracic surgery. Patients after lung cancer resection have not only altered diaphragm function, but also an abnormal elevated position that may cause the compression of vagus nerve and lead to overstimulation and determine abnormal afferents to the brain.

### 2. The role of proprioception

Several psychophysical approaches have pointed to a major role of the muscle spindles in generating proprioceptive input, particularly for sensation of joint position and direction of movement [32]. Previous studies reported that a few muscle spindles exist in a diaphragm, confined in crural region [33–35]. We assume that when the diaphragm is not working correctly, its proprioceptive ability is reduced. We speculate that a decrease in diaphragm length-tension relationship (where decreased length decreases the force of the contraction) during ventilation, resulting from reduced diaphragm movement, creates a state of insufficient irritability of proprioceptors and subsequent inadequate sensory stimuli to provide optimal postural control and balance maintenance.

Diaphragm plays an important role in modulation of intarabdominal pressure (IAP). Limitation of diaphragm excursion leads to insufficient IAP. Holt et al., found that diaphragm receptors can be categorized into three types. Except muscle spindles, the pressure-sensitive mechanoreceptors are also present [36]. It is possible that not enough IAP causes poor proprioceptors stimulation in the crural region and in consequence disturbs the projection of proprioceptive information to the central nervous system. In the present study we demonstrated that restricted diaphragm movement during deep breathing was correlated with longer path length and greater ellipse area what may confirm this hypothesis.

In these considerations one should also take into account the breathing pattern. Belly breathing distends the abdomen forward, which does not offer any resistance to the diaphragms motion and will therefore actually reduce the diaphragm’s ability to contract efficiently. This types of breathing may lead to poor proprioceptive postural strategies.

### 3. The role of diaphragm fatigue

Diaphragm fatigue is a condition in which there is a reduction in the force generating capacity of the muscle resulting from muscle activity underload which is reversible by rest [37]. This fatigue can be due to contractile dysfunction, or to failure of neural activation, which are known as peripheral and central fatigue, respectively [38].

Dual function of a diaphragm (ventilation and postural) is simultaneous. Increased demand on one of its functions (an inspiratory loading task) will inevitably abolish the other function and often reduce the respiratory muscles' ability to perform their postural duties. It was already previously observed by Hodges [39] and Vostatek [40] that if the demand for breathing increases, the role of the diaphragm in low back stability declines. With reference to these, it is reasonable to postulate that diaphragm fatigue may exacerbate balance instability.

One shall not rule out that breathing pattern disorders may also contribute to balance disorders. It has been considered that rapid shallow breathing may reflect the presence of respiratory muscle fatigue [41]. This type of ventilation is observed in chest breathing and is associated with less diaphragm movement. We suppose that diaphragm muscle fatigue has a negative influence upon proprioceptive feedback. It is likely that some proprioceptive signals derived from the diaphragm may be altered by specific fatigue.

### 4. The role of the phrenic nerve

Sensory innervation (proprioception) to the diaphragm is mostly from the phrenic nerves. It can be assumed that if the position of the diaphragm is not physiological, the phrenic nerve is retracted or irritated in different ways, causing improper proprioceptive afference to the CNS.

Our study has some limitations. They mainly concern the differences between clinical and the control group in terms of the subjects age and gender. The first one is the result of difficulties in selecting people over 60 years old, who do not have any medical conditions, especially those that can clearly affect the parameters of body sway and are listed in the exclusion criteria from the study. Therefore, we decided to create control group with students and hospital staff. However, thanks to this it was possible to show that the correlation of the thickness and mobility of the diaphragm with partial parameters of postural swaying occurs not only in people over 50, but also in healthy, young people. With regard to the gender differences, it should be noted that the diaphragm is the main human respiratory muscle, both in women and men. It seems, therefore, that these differences should not affect the results obtained in the present study. In this study, pain rating scale was no used, although pain is one of the main factors affecting the patient's respiratory characteristics in the post-operative stage. All patients undergoing lobectomy received analgesic treatment using oxycodone in a safe dose from an infusion pump (controlled by the patient) on demand, followed by oral analgesics from the third level of the analgesic ladder developed by the World Health Organization. In addition, not all patients undergoing lobectomy started the second ultrasound and stabilometric measurement between 3 and 5 days after the surgery. This was due to the longer time needed to remove the drains, the presence of post-operative complications or the poor psychophysical condition of the patient, which prevented the repeated measurement. This may, therefore, slightly affect the results.

In summary, the diaphragm is mainly recognized as the primary muscle responsible for breathing. In recent years the diaphragm has gained interest for its involvement in non-ventilatory functions, i.e. postural function, where the diaphragm precedes any movement of the body by lowering and subsequently establishing abdominal pressure which helps to stabilize the lumbar part of the spine. In this study we documented a new function of the diaphragm which refers to static balance maintenance, and we described possible explanations for this phenomenon. The results of the study presented here may have clinical significance not only for medicine but also for rehabilitation, which uses a number of different methods in equilibrium training. The question whether diaphragm muscle training affects the improvement of balance parameters still remains unsolved, but there is already some indirect evidence. Janssens et al., found that inspiratory muscle training (60% of maximal inspiratory pressure) improve proprioceptive postural control by addressing the trunk stabilizing function of the diaphragm. The authors conclude that an increase of inspiratory muscle strength may enable individuals to reactivate proprioceptive signals and to switch to a more optimal proprioceptive strategy [42] However, further studies are needed in this topic.

## Conclusions

The main conclusion drawn from this study is that the diaphragm muscle plays an essential role in static balance maintenance, and like previously described others functions, this balance function is also inextricably linked to breathing. Impairment of diaphragm function manifested by the decrease in muscle thickness and movement restriction is strongly associated with balance disorders in a clinical sample and among healthy subjects. The following detailed conclusions summarize the findings of the conducted study:

1. Greater diaphragm thickening during active breathing, which reflects the magnitude of diaphragmatic effort (similarly to an ejection fraction of the heart), is related to better static balance parameters.
2. Lower values of diaphragm thickness fraction (DTF) index, which depend on diaphragmatic activity and reflect the diaphragm work of breathing, are associated with greater balance deficits. Values of DTF less than 20% interpreted as diaphragm atrophy or diaphragm paralysis are significantly attributable to balance disturbance. This leads us to a hypothesis that the decrease in muscle thickness may play a significant role in etiology of static balance disorders.
3. Better diaphragm motion during quiet breathing is linked to lower balance instability. The same, but stronger trend is observed during deep breathing, which suggests that the diaphragm's ability to create optimal abdominal pressure is associated with balance stability.
4. There is no correlation between diaphragm muscle excursion during sniff maneuvers and balance parameters. This suggests that the strength of primary respiratory muscles have no impact on postural sways in upright position.
5. Deterioration of diaphragm function observed after thoracic surgery is closely related to deterioration of balance maintenance.
